# Mechanistic Insights into the Antimicrobial Effect of Benzodioxane-Benzamides Against *Escherichia coli*

**DOI:** 10.3390/antibiotics15020126

**Published:** 2026-01-27

**Authors:** Lorenzo Suigo, Alessia Lanzini, Valentina Straniero, William Margolin

**Affiliations:** 1 Department of Microbiology and Molecular Genetics, McGovern Medical School, University of Texas, Houston, TX 77030, USA; lorenzo.suigo@uth.tmc.edu; 2 Dipartimento di Scienze Farmaceutiche, Università degli Studi di Milano, Via Luigi Mangiagalli, 25, 20133 Milano, Italyvalentina.straniero@unimi.it (V.S.)

**Keywords:** antibiotics, FtsZ, bacterial cell division, benzodioxane-benzamides, antibiotic resistance mechanisms, FtsW

## Abstract

**Background/Objectives**: The bacterial cell division machinery is emerging as an attractive target for antimicrobial compounds. FtsZ, a highly conserved essential division protein, is the target for a number of small molecules such as benzamides. Recent studies show that benzodioxane-benzamides (BDOBs) are among the most potent inhibitors of FtsZ function in Gram-positive bacteria, although their ability to inhibit Gram-negative FtsZ, in particular *Escherichia coli* FtsZ, has been more controversial. **Methods**: Here, we use genetic and cytological methods to demonstrate that FtsZ of efflux pump-disabled *E. coli* can be efficiently targeted by BDOBs. **Results**: We show that engineered mutants and spontaneous variants map in or near the interdomain cleft (IDC) of FtsZ that confers resistance to BDOBs, similar to previous results with Gram-positive FtsZs. We also uncover spontaneous extragenic mutants that can confer high levels of resistance to at least one potent BDOB, including a mutant that encodes a novel hyperfission variant of the essential cell division protein FtsW. **Conclusions**: Our evidence indicates that as with Gram-positive bacteria, the IDC of Gram-negative bacterial FtsZ is directly targeted by BDOBs, provided efflux pumps are disabled. We also conclude that FtsZ-independent factors can influence the effect of BDOBs on *E. coli* cell division, including activation of division septum synthesis.

## 1. Introduction

The current health emergency related to the problem of antimicrobial resistance (AMR) is widely known among the scientific community. In 2019, more than 4.95 million deaths were associated with AMR [1,2,3]. The reasons behind the spreading of AMR include the misuse of antibiotics (not limited to human use) and the slow development of novel antibacterial molecules. Currently, carbapenem-resistant Enterobacterales (CREs), third-generation cephalosporin-resistant Enterobacterales (3GCREs), and carbapenem-resistant *Acinetobacter baumannii* (*CRAB*) [4], all Gram-negative bacteria, are categorized in the critical priority group by WHO. Consequently, the development of novel therapeutics that can interfere with multi-drug-resistant strains remains of absolute importance. More specifically, small molecules that interfere with unexploited essential bacterial processes stand as the ideal strategy to overcome the current mechanisms of resistance [5]. Among these are inhibitors of bacterial cell division, with one agent (TXA709) currently in Phase 1 development as an anti-MRSA agent [6].

Bacterial cell division is a well-orchestrated process essential for bacterial colonization and proliferation. Most of the proteins involved in this process are known, with FtsZ widely recognized for having a key and central role. This tubulin-like protein acts at the initial stages by assembling into polymers at the center of the cell, forming the so-called “Z-ring” or “proto-ring” [7]. In *E. coli*, this proto-ring also includes the FtsA and ZipA proteins, which are required to tether FtsZ polymers to the cytoplasmic membrane [8]. The proto-ring serves as a guide for the recruitment of additional cell division proteins, which ultimately assemble into the divisome complex [9]. The divisome includes FtsW and FtsI proteins, which catalyze the synthesis of septal peptidoglycan necessary for cytokinesis.

**Figure 1:**
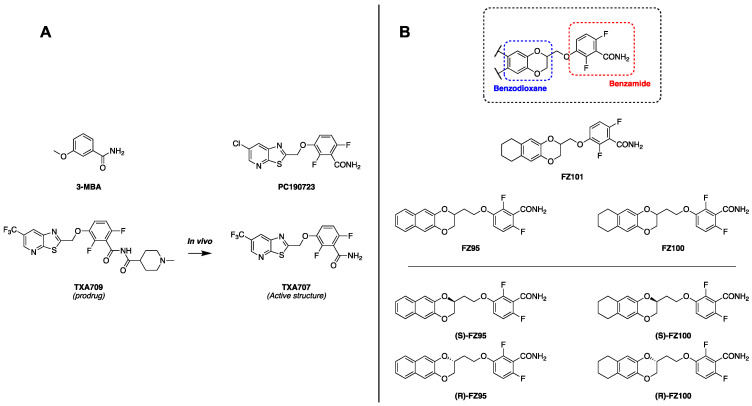
Structures of previously characterized benzamides, including those relevant for this study. (**A**) Structures of the most relevant benzamides: 3-methoxybenzamide (3-MBA) and PC190723 represent two of the first important derivatives discovered. TXA707, with its prodrug TXA709, is the first benzamide that has reached phase 1 clinical trials. (**B**) General structure of benzodioxane-benzamides (BDOBs) and most important derivatives. Below are represented the enantiomeric pure forms FZ95 and FZ100.

Disruption of FtsZ assembly rapidly arrests cell division, which in rod-shaped bacilli such as *E. coli* results in cell filamentation and death [10,11,12]. Over the last two decades, many studies have sought to develop antibacterial agents that targeted FtsZ. Several chemical classes were identified, including zantrins [13], berberine analogues [14], quinolinium compounds [15], chrysophaentins [16], GTP analogues [17], and the most studied class of molecules, benzamides. The development of benzamides as antibacterials started with 3-methoxybenzamide (Figure 1A), leading to the isolation of the aforementioned TXA709, a prodrug of TXA707 (Figure 1A). This compound was developed while attempting to enhance the pharmacokinetic properties of PC190723 (Figure 1A), a well-known reference benzamide compound, also characterized by high potency against MRSA [18]. In addition to these promising compounds, several subclasses of benzamides were developed, including benzodioxane-benzamides (BDOBs), in which the planar aromatic moiety of TXA707 is substituted by a benzodioxane ring (Figure 1B). In the last decade, the development of this subclass led to anti-staphylococcal compounds, such as FZ95 and FZ100 (Figure 1B), with potencies ranging from 4 to 10 times stronger than TXA707-9 against both MSSA and MRSA [19].

Although FtsZ is highly conserved among most bacterial species, the ability of benzamides to interfere with Gram-negative bacterial growth remains controversial. Indeed, PC190723, TXA707, FZ100, and most of the benzamides are completely inactive against wild-type *E. coli* [18,20,21,22,23]. However, it was reported that disrupting outer membrane permeability of *E. coli* allows PC190723 to inhibit cell growth and division [24]. Surprisingly, in vivo evidence indicated that this inhibition did not seem to act through FtsZ, raising the possibility of non-FtsZ resistance mechanisms. Moreover, Poddar et al. recently suggested that the inability of benzamides to interact with *E. coli* FtsZ could be due to several salt bridges within the protein that impede drug access [25,26]. These salt bridges are absent in *Staphylococcus aureus* and *Bacillus subtilis* FtsZs, which are highly susceptible to PC190723. Nonetheless, Bryan and colleagues recently reported that selected bromo-oxazole benzamides can directly interact with *A. baumanii* FtsZ, promoting an antimicrobial effect, showing how membrane permeability is the main limiting factor for that particular Gram-negative species [27]. This conundrum prompted us to investigate the mechanism of cell division inhibition by benzamides in outer membrane-compromised *E. coli*.

Recently, after an in-depth evaluation of MICs of BDOBs in Δ*acrAB* or Δ*tolC* mutant *E. coli* strains, we were able to demonstrate that many BDOBs are able to interfere with *E. coli* FtsZ assembly in vitro and confirmed that the inability of BDOBs to exert antimicrobial activity in vivo is due to other factors such as the presence of efflux pumps [11]. Even if these recent findings ushered in a way to exploit these potential novel tools to address Gram-negative bacterial infections, the pharmacodynamics of BDOBs, well-established in Gram-positives, are still significantly underexplored in Gram-negative bacteria. In the present work, we sought to close this gap by demonstrating whether BDOBs directly target FtsZ in *E. coli* cells, which residues of FtsZ might be involved, and if other non-FtsZ factors play a role in the ability of BDOBs to disrupt cell division.

## 2. Results

### 2.1. PAβN Restores Antimicrobial Activity of BDOBs Against WT E. coli

We recently reported that BDOBs can inhibit cell division in *E. coli* mutants ∆*tolC* or ∆*acrAB*, which are deficient for RND efflux pumps [11,21]. Nonetheless, as a foundation of this study, we wanted to confirm that chemical inhibition of efflux pumps is effective in restoring BDOB activity against *E. coli*, as previously reported for other benzamides [12]. As shown in Table 1, co-administration of phenylalanine-arginine beta-napthylamide (PaβN) [28] with two previously reported potent BDOBs, (*S*)-FZ95 or FZ101 [11,21], restored the bactericidal activity of these compounds, with MIC values equal to those observed against *E. coli* Δ*tolC* mutants. These data confirm that RND efflux pumps play a key role in impeding the activity of BDOBs and how their inhibition—either chemically or genetically—is sufficient to restore complete activity.

### 2.2. Spontaneous FZ101-Resistant Mutants Map to the E. coli FtsZ Interdomain Cleft (IDC)

To exert its action in *S. aureus*, PC190723 and most known benzamides interact through their benzamide moiety with key residues in the Interdomain Cleft (IDC) of FtsZ, a subdomain that separates the major conserved N-terminal and C-terminal domains of the protein [10]. These residues include, among others, V207, L209, and N263, in *S. aureus* [18,22,29]. To observe if the binding site is conserved in *E. coli* FtsZ (and likely, in other Gram-negative species), we screened for spontaneous genomic mutants resistant to the BDOB FZ101, using a ∆*tolC* strain of *E. coli* (WM6922).

**Figure 2:**
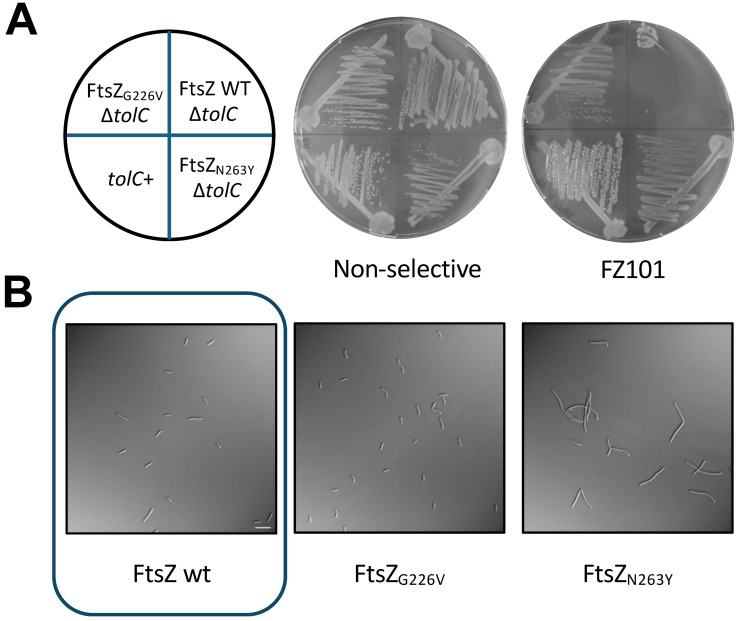
FZ101 spontaneous resistant mutants encode single residue changes in the *E. coli* FtsZ IDC. (**A**) *E. coli* strains WM1074 (WT), WM6922 (Δ*tolC*), WM6922 (FtsZ_G226V_), and WM6922 (FtsZ_N263Y_) were streaked onto LB + 20 μM FZ101 or non-selective LB agar. Plates were incubated at 30 °C for 18 h prior to visualization; (**B**) DIC microscopy images of isolated WM6922 (∆*tolC*) resistant strains expressing FtsZ_G226V_ and FtsZ_N263Y_ from the native *ftsZ* locus. Although both FtsZ variants confer resistance to FZ101, only FtsZ_N263Y_ shows perturbed FtsZ functionality. The image of WT control cells is boxed. Scale bar = 10 μm.

We isolated two mutants that were able to form colonies on agar plates containing >20 μM FZ101 as well as non-selective plates (Figure 2A). Genome sequencing of their *ftsZ* genes revealed single missense mutations that encoded variants G226V and N263Y, respectively. As shown in Figure 2B, while allowing growth, the N263Y variant resulted in morphological defects, such as branching and partial filamentation, consistent with altered FtsZ function.

**Figure 3:**
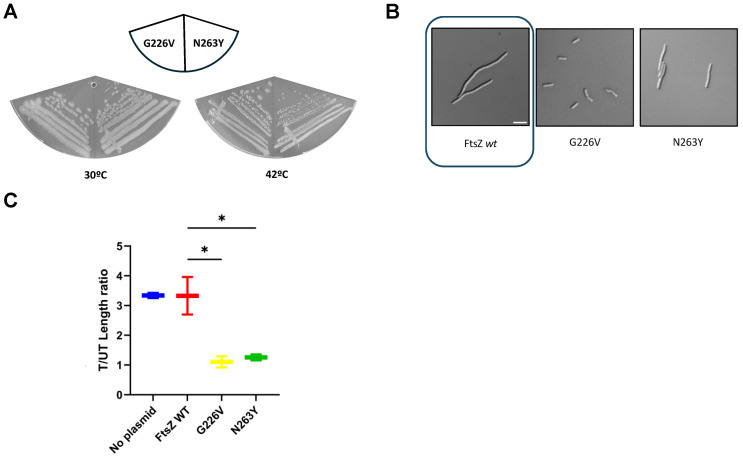
Spontaneous genomic variants FtsZ_G226V_ and FtsZ_N263Y_ confer resistance to selected benzamides. (**A**) To confirm the functionality of these variants, mutations encoding G226V and N263Y were introduced into pJSB100-FtsZ, transformed into the *ftsZ84*(ts) strain WM5188, and tested for their ability to complement the thermosensitive allele at the nonpermissive temperature of 42 °C. (**B**) In addition, to also confirm how the G226V and N263Y amino acid changes confer resistance, pJSB100-FtsZ_G226V_ and pJSB100-FtsZ_N263Y_ were transformed into WM6922 (∆*tolC*) and evaluated as described in the main text. Cells in the presence of FZ101 are shown. The image of WT control cells is boxed. Scale bar = 5 μm. (**C**) Cell length measurements of the cultures in (**B**) are shown as treated/untreated (T/UT, defined as the ratio between average lengths in the presence of FZ101 vs. the average lengths in the presence of DMSO). * = *p* < 0.05; CI = 95%; N ≥ 75 cells from 2 independent cultures.

To explore this further, we cloned the mutant *ftsZ* genes into the pJSB100 plasmid, which expresses *ftsZ* or *ftsZ* mutant alleles under arabinose control and tested their ability to function in an *ftsZ84* thermosensitive strain at the nonpermissive temperature of 42 °C. Whereas pJSB100-FtsZ_G226V_ completely complemented at 42 °C, pJSB100-FtsZ_N263Y_ was only able to partially complement, resulting in weaker growth (Figure 3A). Importantly, both plasmids were able to support growth in the presence of 15 μM FZ101, strongly suggesting that the G226V or N236Y residue changes in FtsZ are sufficient to confer benzamide resistance (Figure 3B). Indeed, cells expressing FtsZ_G226V_ or FtsZ_N263Y_ instead of WT FtsZ and treated with 15 μM FZ101 had no significant length differences compared to cells treated with a corresponding volume of DMSO carrier, whereas cells expressing WT FtsZ exhibited a 3.5-fold length increase (Figure 3C, treated/untreated cell length ratios).

To observe if these mutations confer resistance to other BDOBs, we tested the original FZ101-resistant strains in the presence of (*S*)-FZ95, the eutomer of FZ95, a previously characterized naphthodioxane-benzamide [11,19]. To do so, we spotted serially diluted cells of the FZ101 resistant strains onto LB plates containing 0.25 μg/mL (*S*)-FZ95. As shown in Figure S1, the parent strain WM6922 was not viable under these conditions as expected, but the *ftsZ_G_*_226*V*_ strain was fully resistant to (*S*)-FZ95. The *ftsZ_N_*_263*Y*_ strain, which has a growth defect even without added compound (see also Figure 3), nevertheless was partially resistant to (*S*)-FZ95 as well (Figure S1).

The N263 residue is conserved among *S. aureus*, *B. subtilis*, and *E. coli* FtsZ, maps to the IDC, and variants of this residue are well known to confer resistance in other microorganisms such as *S. aureus,* especially N263K [30]. In contrast, variants at G226 were not reported in other investigated species. Its position with respect to the IDC and other known benzamide-interacting residues suggests that G226 is probably not directly involved in drug binding, but bulkier variants of it could impede access to the drug, similar to the G196A variant in *S. aureus* [30]. These findings indicate that the IDC site is also conserved as the benzamide binding site in Gram-negative species such as *E. coli*, as initially hypothesized.

### 2.3. Variants in the IDC of E. coli FtsZ Can Affect Functionality and BDOB-Resistance

To further strengthen these initial findings with spontaneous mutants, we engineered specific variants within the *E. coli* FtsZ IDC and evaluated their phenotypes. We targeted 4 amino acids: N263, V208, M206, and G195. The first 3 are likely directly involved in the binding to the drug, whereas variants at G195, as mentioned above, are known to block drug access to the binding site in other species, thus preventing its activity [30]. Before evaluating the potential drug-resistant phenotype of the variants, their functionality was assessed by introducing pJSB100-FtsZ expressing each variant into the *E. coli ftsZ84*(ts) background. Cells were grown at permissive temperature until the early exponential phase and then switched to nonpermissive temperature for 1 h prior to visualization (also see Section 4).

**Figure 4:**
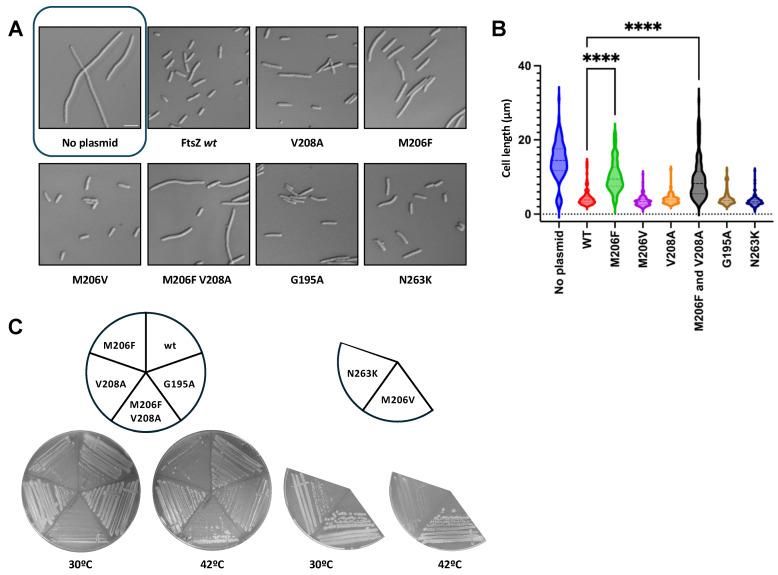
Directed residue changes in the IDC can affect *E. coli* FtsZ functionality. (**A**) Mutations encoding all the engineered variants were introduced into pJSB100-FtsZ, transformed into the ∆*tolC ftsZ84*(ts) strain WM5188, and evaluated for complementation as described in the main text, in the absence of FZ101. DIC images of cells of all the evaluated strains are shown, with a box around the image of control cells lacking a plasmid. Scale bar = 5 μm. (**B**) Cell lengths were measured and plotted. **** = *p* < 0.0001; CI = 95%; N ≥ 75 cells from 2 independent cultures. (**C**) Growth on solid media of WM5188 transformed with the mutated plasmids, cultured at 30 °C and 42 °C.

As expected, pJSB100-FtsZ WT was able to complement at a nonpermissive temperature, with normal-length cells, whereas untransformed cells were significantly filamented (Figure 4A). We observed similar behavior with pJSB100-FtsZ M206V, V208A, and G195A, suggesting that these mutants exhibit full or nearly full functionality (Figure 4A,B). In contrast, although their lengths were normal, cells expressing FtsZ_N263K_ had significantly altered morphologies: partially filamentous cells as well as several minicells were observable (Figure 4A), similarly to what was seen previously with N263Y. Additional variants of M206, such as M206F, led to a non-functional protein and consequent filamented cells. We also found that adding an FZ101-resistant residue change (V208A) to FtsZ_M206F_ was unable to rescue M206F’s lack of functionality at the restrictive temperature (Figure 4A,B). A similar experiment was conducted on solid media, where the same cultures were grown at both permissive and restrictive temperatures (Figure 4C). Considering its lack of functionality, we did not study M206F further.

To evaluate if these variants could confer a BDOB-resistant phenotype, the same pJSB100 plasmids were transformed into the *E. coli* Δ*tolC* strain (WM6922) and evaluated as described in the Section 4. Briefly, cells were grown to the early exponential phase, and then 15 μM FZ101 or a corresponding volume of DMSO was added to the cultures, which were then grown for an additional hour prior to examining by microscopy. Under these conditions, FtsZ expressed from the native *ftsZ* locus was inhibited by BDOB, which tested the ability of the arabinose-induced FtsZ variants from the pJSB100 plasmids to promote cell division or not, depending on the variant’s susceptibility to the drug.

**Figure 5:**
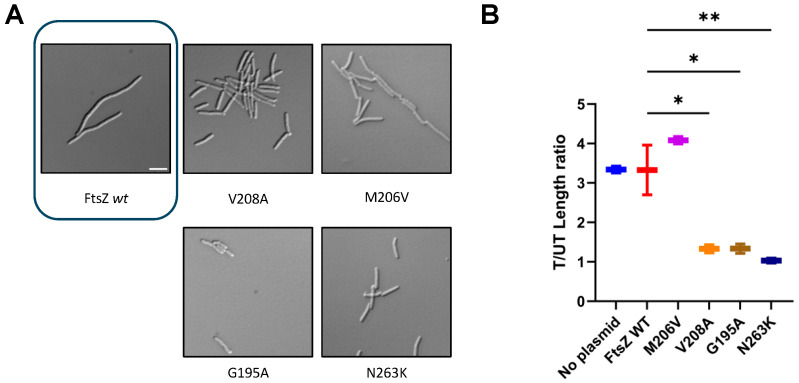
Directed residue changes in the IDC achieve resistance to selected benzamides. Plasmids expressing mutated FtsZs described in Figure 4 were transformed into the ∆*tolC* strain WM6922. Each strain was cultured at 30 °C with 0.2% arabinose until OD values of 0.2–0.3. Then, 15 µM FZ101 or an equal volume of DMSO was added, and the strain was cultured to OD 0.6–0.8 prior to microscopy imaging. (**A**) DIC microscopy images of cells expressing selected FtsZ variants in the presence of FZ101. The image of WT control cells is boxed. (**B**) Cell lengths were measured and reported as treated/untreated (T/UT) ratios. Scale bar = 5 µm; ** = *p* < 0.01; * = *p* < 0.05; N ≥ 75 cells from 2 independent cultures.

As shown in Figure 5A, cells transformed with pJSB100-FtsZ WT rapidly filamented in the presence of FZ101. Similarly, as observed by both imaging (Figure 5A) and cell length measurements (Figure 5B), FtsZ_M206V_ did not confer resistance, whereas FtsZ_G195A_ and FtsZ_V208A_ conferred partial resistance, with treated/untreated cell length ratios ranging from ~1.3–1.5 along with minor cell morphology defects. FtsZ_N263K_ had a completely resistant profile, although this variant again exhibited morphological alterations, which remained unchanged in presence of FZ101. All the engineered strains were also tested for cross-resistance to 0.25 μg/mL (*S*)-FZ95 in both the presence and absence of 0.2% arabinose. Consistent with levels of resistance to FZ101, induced levels of FtsZ_M206V_ showed the lowest level of resistance, equivalent to WT FtsZ, whereas the other variants exhibited higher levels of partial resistance (Figure S2). These data further strengthen the hypothesis that the target of BDOBs in *E. coli* is FtsZ, specifically in the IDC.

### 2.4. Non-FtsZ Mutations Can Promote Resistance Towards Selected BDOBs

The isolation of spontaneous mutants resistant to (*S*)-FZ95 on solid media was more challenging than for FZ101, as the selective effect of the drug in plates seemed to be significantly lower for reasons that are unclear. Nonetheless, while performing MIC assays to evaluate new BDOB derivatives active on *E. coli* Δ*tolC* strains, we noticed that some cultures were able to grow in liquid media at 15X MIC and others at 30X MIC concentrations of (*S*)-FZ95. This suggested that these cultures potentially harbored mutants that acquired resistance to (*S*)-FZ95 and prompted us to study them further.

**Figure 6:**
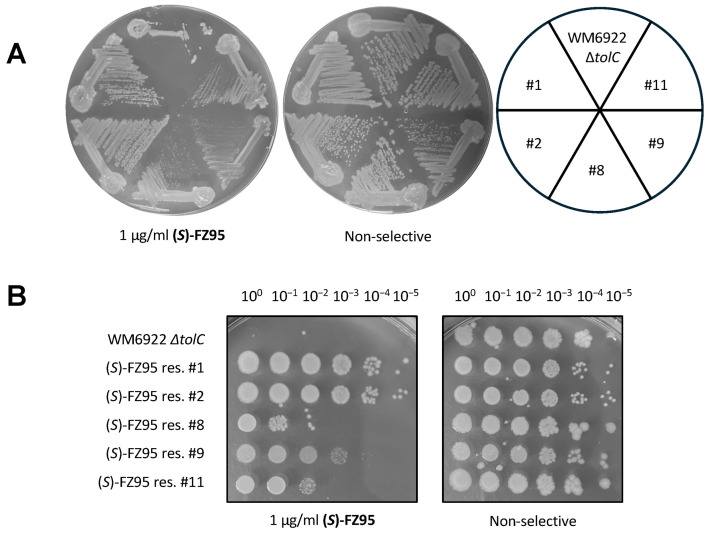
(*S*)-FZ95 spontaneous resistant strains exhibit different degrees of (*S*)-FZ95 resistance. (**A**) Selection for growth of the ∆*tolC* strain WM6922 on LB plates containing 1 μg/mL (*S*)-FZ95 yielded 5 independent colonies that were able to maintain partial to full growth after streak-purifying. (**B**) The 5 candidate resistant strains and the WM6922 parent were serially diluted onto 1 μg/mL (*S*)-FZ95 or non-selective plates and incubated at 37 °C for 24 h to quantitate their degree of resistance.

Once isolated, 5 of the cultures were able to grow in liquid media at concentrations of 1 μg/mL (*S*)-FZ95 (2X MIC) and were therefore plated, together with the ∆*tolC* parent strain WM6922, onto compound-containing and non-selective plates (Figure 6A). We named these strains #1, #2, #8, #9, and #11. All 5 showed some ability to grow in the presence of (*S*)-FZ95, but to better quantify their resistance, we spotted serially diluted cells onto plates containing 1 μg/mL (*S*)-FZ95. From this initial evaluation, it was clear that #1 and #2 showed complete resistance, whereas #8, #9, and #11 were partially inhibited by the compound. Unexpectedly, sequencing of the *ftsZ* genes of these isolates revealed no mutations, indicating that mutations in loci other than *ftsZ* may contribute to (*S*)-FZ95-resistance. This prompted us to characterize the phenotype of these (*S*)-FZ95 resistant strains and investigate the genetic alterations responsible for this resistant phenotype, ultimately focusing on strains #1 and #2 that displayed the highest levels of resistance. (Figure 6B).

**Figure 7:**
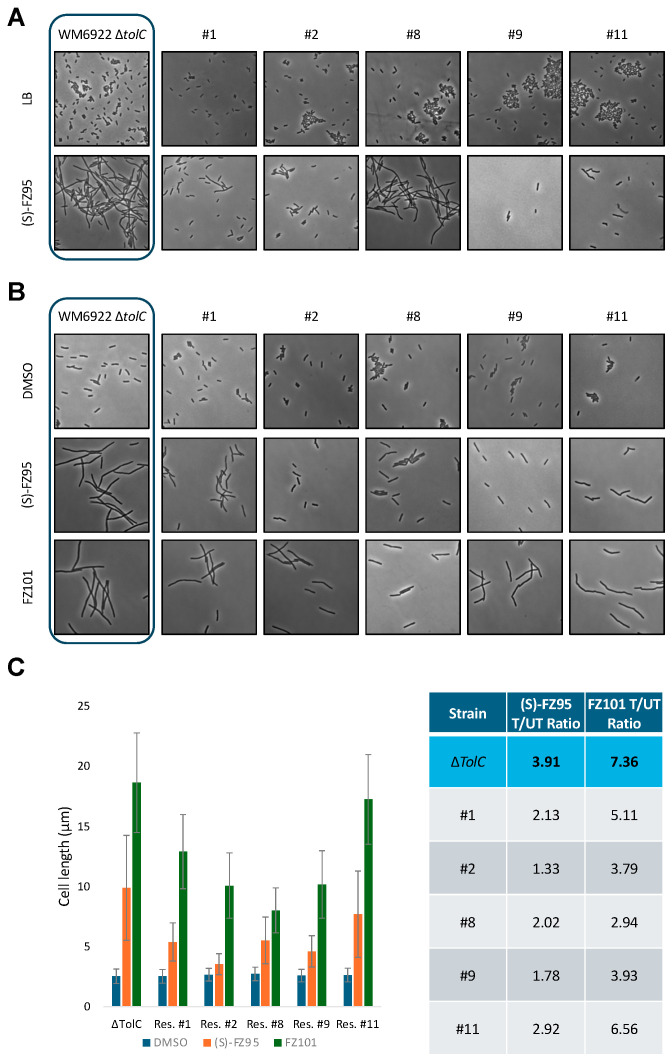
(*S*)-FZ95 spontaneous resistant strains exhibit different degrees of cell aggregation and filamentation in response to (*S*)-FZ95 or FZ101. (**A**) WM6922 (∆*tolC*) and its 5 (*S*)-FZ95-resistant derivatives were cultured overnight in LB ± 1 μg/mL (S)-FZ95 prior to imaging by phase contrast microscopy. Representative images are shown, with images of the parent ∆*tolC* cells boxed. Scale bar = 5 μm. (**B**) The same strains were cultured twice at 30 °C until reaching the early-log phase (OD_600_ = 0.2/0.25). Each strain was then exposed to either 1 μg/mL (*S*)-FZ95, 15 µg/mL FZ101, or a corresponding volume of DMSO. Cells were grown for 1.5 h prior to imaging by phase contrast microscopy. Representative images are shown, with images of the parent ∆*tolC* cells boxed. Scale bar = 5 μm. (**C**) Cell lengths are represented as treated/untreated ratios (T/UT), defined as the ratio between the mean cell lengths in the presence or absence of each BDOB inhibitor; N ≥ 75 cells from 2 independent cultures. Given the qualitative nature of the comparison, no statistical analysis was performed.

We initially incubated all 5 resistant strains plus the parent strain overnight in LB ± 1 μg/mL (*S*)-FZ95, followed by OD measurements and microscopy of the saturated cultures. As expected, *E. coli* Δ*tolC* (WM6922) cells were filamentous in the presence of (*S*)-FZ95 but were normal in shape and length in the absence of the compound. In contrast, the 5 examined strains exhibited different degrees of resistance to (*S*)-FZ95: strains #1 and #2 showed nearly identical cell morphologies in the presence or absence of the compound, with very little to no filamentation (Figure 7A). Consistent with their partial resistance phenotypes, cells of strains #8 and #11 divided less well in the presence of the compound than #1 and #2 but still better than the parent strain. The growth of strain #9 in liquid media was significantly affected by the presence of the compound (Table S1), although surviving cells in culture exhibited normal lengths (Figure 7A). Interestingly, cells of all resistant or partially resistant strains showed a similar significant tendency to clump in plain LB, although the reproducibility of this phenomenon and its correlation with the resistance phenotype were unclear (Figure 7A).

Having obtained data for cells that reached high density, we subsequently tested the degree of resistance when the benzamide inhibitor was added to early-log cells. To do so, all the resistant strains and the parent strain were evaluated using the same protocol described above (see Section 4): cells were cultured at 30 °C until OD = 0.2/0.25, followed by addition of 1 μg/mL (*S*)-FZ95 or a corresponding volume of DMSO and incubated for an additional 1.5 h prior to final OD measurements and microscopy visualization. As expected, cells of the parent strain WM6922 significantly filamented, with a T/UT cell length ratio of ~4. Strain #2 had a T/UT cell length ratio of ~1.5, consistent with its higher level of resistance. All the other strains presented intermediate degrees of resistance, with T/UT ratios ranging from ~1.8 to 3 and slightly altered cell morphology (Figure 7B,C).

To determine whether the resistance profile was conserved across different BDOBs, in the same experimental setting, cells were also cultured with 7.5 μg/mL FZ101 (2X MIC) prior to final OD measurement and microscopy. Interestingly, the parent strain and all the resistant strains were more susceptible to FZ101 than to (*S*)-FZ95 (Figure 7B). *E. coli* Δ*tolC* significantly filamented upon treatment with FZ101, with cell length values 7.5 times higher than that of the DMSO-treated culture (Figure 7C). All the strains evaluated were less susceptible to FZ101 than the parent strain, with T/UT ratios ranging from 3 to 6.5 (Figure 7C).

### 2.5. Investigating Potential Mechanisms Behind Non-FtsZ Resistance to BDOBs

We next investigated which mutations might be the basis for the resistance to BDOBs, focusing on the most resistant strains, #1 and #2. Interestingly, whole genome sequencing of #1 and #2 revealed a single guanine deletion in position 131 of *yidE*, resulting in a frameshift of the last 7/8th of the gene. The encoded YidE protein is a member of the Aspartate: Alanine Exchanger family, the physiological role of which has not yet been completely elucidated [31]. Strain #1 also harbored a 1342 bp insertion in *sanA*, another poorly characterized gene, whereas #2 harbored a SNP within *ftsW*, which encodes an essential and highly conserved glycosyl transferase that synthesizes the cell division septum along with the FtsI transpeptidase [32]. The SNP (A to T at nucleotide 812) results in the expression of the variant FtsW_Q221L_ (Table 2). Q221 is located within a predicted short cytoplasmic loop that connects transmembrane domains 6 and 7 of the 10 transmembrane domains of FtsW.

**Figure 8:**
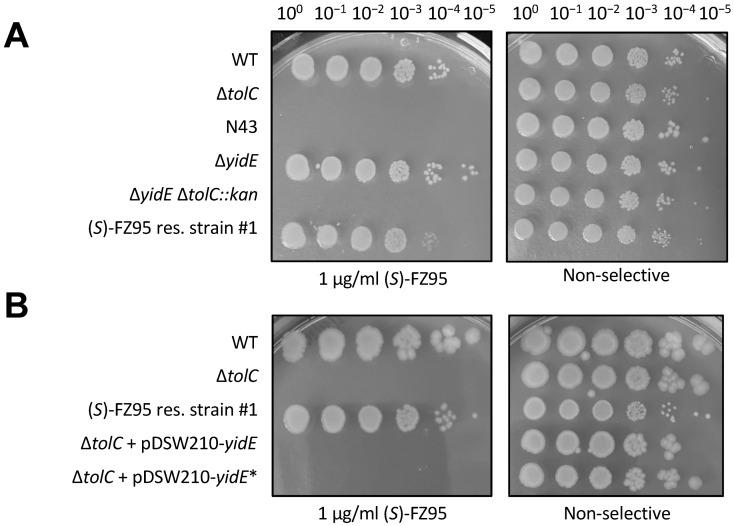
Deletion or overexpression of *yidE* does not promote (*S*)-FZ95 resistance. (**A**) The Δ*yidE::kan* allele was introduced into the WT WM1074 strain as described in the main text. The efflux pump-defective strain WM6794 (*E. coli* strain N43) was included as an additional control for drug sensitivity. WM1074 Δ*yidE tolC*+ was included as a negative control. (**B**) To test if the expression of mutated *yidE* in resistant strains #1 and #2 (hereafter named as yidE*) could be responsible for the phenotype, *yidE* and yidE* were cloned in the plasmid pDSW210 and transformed in the Δ*tolC* strain WM6922. For both experiments, mid-logarithmic phase cultures were grown at 37 °C, serially diluted, and spotted onto LB agar ± 1 μg/mL (*S*)-FZ95. Plates were incubated at 37 °C overnight.

As the same identical *yidE* mutation is present in the two strains most resistant to (*S*)-FZ95, and assuming that the frameshift mutation is equivalent to a loss-of-function of YidE, our initial hypothesis was that *yidE* plays a key role in allowing (*S*)-FZ95 to exert its action against Δ*tolC E. coli* and, therefore, its deletion should confer resistance to the compound. Consistent with this idea, *yidE* was WT in strain #9, which showed higher susceptibility to (*S*)-FZ95. To test this hypothesis, we introduced a ∆*yidE::kan* deletion into a WT *E. coli* strain (WM1074) by P1 phage transduction from a Keio strain [33]. After removing the Kan resistance cassette by FLP recombination using pCP20, we introduced the ∆*tolC::kan* allele through a second P1 phage transduction. Unexpectedly, this de novo engineering of Δ*yidE* into a fresh Δ*tolC* background was not sufficient to reproduce the resistant phenotype (Figure 8A). Similarly, neither overexpression of the mutated *yidE* gene in a Δ*tolC* background strain (Figure 8B) nor reconstitution of WT *yidE* in the (*S*)-FZ95 resistant strain #1 were sufficient to restore susceptibility to (*S*)-FZ95. These results suggest that the frameshift at the *yidE* locus in the (*S*)-FZ95-resistant strains has no obvious role in resistance, and we did not pursue this further.

**Figure 9:**
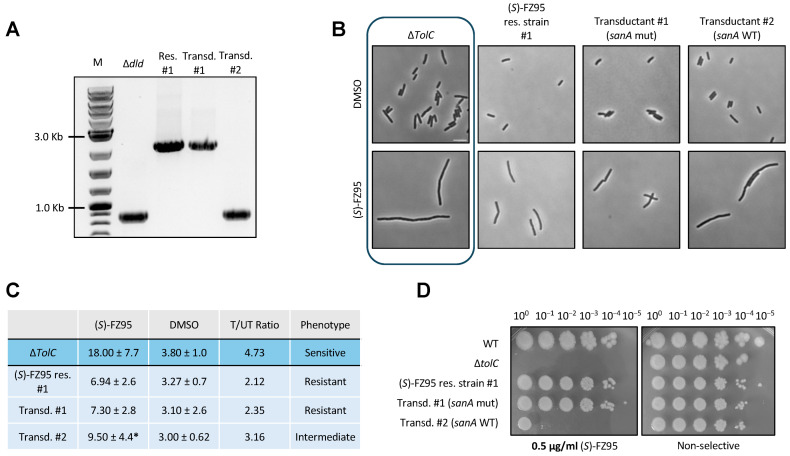
Reconstitution of the WT *sanA* allele in (*S*)-FZ95 res. strain #1 only partially restores (*S*)-FZ95 susceptibility. (**A**) Restoration of the WT *sanA* locus by P1 cotransduction with *∆dld::kan*. Agarose gel electrophoresis of the PCR-amplified genomic *sanA* gene (primers 2974 + 2975, see Supplementary Materials), confirming the presence of WT *sanA* in the otherwise WT ∆*dld::kan* strain (small DNA fragment) and replacement of the mutant *sanA* gene (large fragment) present in FZ95-resistant strain #1 with the WT *sanA* gene from the *∆dld::kan* strain (transductant #2, small DNA fragment). Transductant #1 retains the mutant *sanA* gene (large DNA fragment). (**B**) *E. coli* Δ*tolC* (WM6922), (*S*)-FZ95 res. strain #1 (LS17), *dld::kan* transductant #1 (LS26), and *dld::kan* transductant #2 (LS27) were cultured twice at 30 °C until early-log phase (OD_600_ = 0.2/0.25), then one culture per strain was treated with 0.5 μg/mL (*S*)-FZ95 and the other with a corresponding volume of DMSO. Cells were grown for 1.5 h prior to imaging by phase contrast microscopy. Representative images are shown, with images of the parent ∆*tolC* cells boxed. Scale bar = 5 μm. (**C**) Cell lengths of the microscopy evaluation are shown and expressed as mean ± sdv. T/UT = treated/untreated; * = *p* < 0.05 (S-FZ95 res. #1 used as reference); N ≥ 75 cells from 2 independent cultures. (**D**) Mid-logarithmic phase cultures were grown at 37 °C, serially diluted, spotted onto LB plates ± 1 μg/mL (*S*)-FZ95, and incubated at 37 °C overnight.

After ruling out the potential contribution of *yidE*, we next investigated whether the modification of the *sanA* locus in resistant strain #1 could be the main factor in conferring resistance. If this was the case, then replacing the mutant *sanA* allele with the WT *sanA* allele should be sufficient to restore susceptibility. To test this idea, we introduced the WT *sanA* allele via P1 cotransduction of a linked Δ*dld::kan* marker into strain #1. We first converted strain #1 into a kanamycin-sensitive recipient through excision of the Kan resistance cassette portion of the *∆tolC::kan* marker in strain #1 via FLP recombination, using pCP20. We then proceeded with the Δ*dld::kan sanA+* transduction, expecting a ~75% cotransduction frequency based on the ~10 kb distance between the *dld* and *sanA* loci. We screened for either retention of mutated *sanA* (LS26) or conversion to WT *sanA* (LS27) by PCR amplification of the *sanA* locus and detection of the large size difference between the two amplicons on an agarose gel (Figure 9A).

As shown in Figure 9B, insertion of WT *sanA* had a moderate effect on the (*S*)-FZ95 resistance profile of strain #1, with the ratio of treated vs. untreated cells changing from ~2 to ~3 with WT *sanA* wt whereas the fully susceptible parent strain has a value of ~4.75 (Figure 9C). Consistent with this, spot dilution plates showed that the restoration of WT *sanA* to strain #1 resulted in several logs of viability loss, although not a complete loss as with the parent strain WM6922. These results suggest that the altered *sanA* locus is only partially responsible for mediating (*S*)-FZ95 resistance in #1.

**Figure 10:**
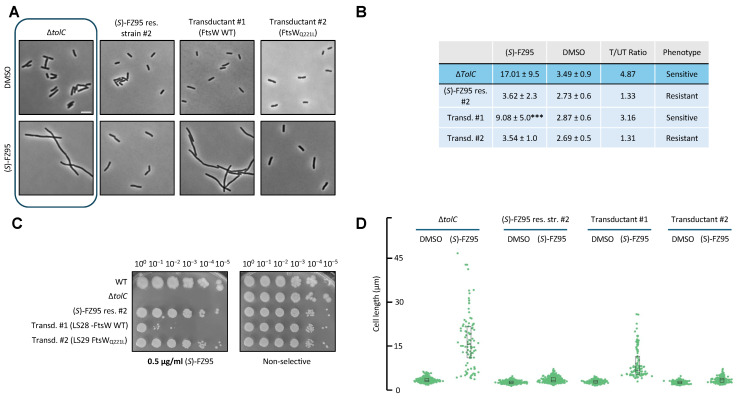
Reconstitution of the WT *ftsW* allele in (*S*)-FZ95 resistant strain #2 restores (*S*)-FZ95 susceptibility. (**A**) As previously described, *E. coli* Δ*tolC* (WM6922), (*S*)-FZ95 res. strain #2, *ilvI::kan* transductant #1, and *ilvI::kan* transductant #2 were cultured twice at 30 °C until early-log phase (OD_600_ = 0.2/0.25), then one culture per strain was treated with 0.5 μg/mL (*S*)-FZ95 and the other with a corresponding volume of DMSO. Cells were grown for 1.5 h prior to imaging by phase contrast microscopy. Representative images are shown, with images of the parent ∆*tolC* cells boxed. Scale bar = 5 μm. (**B**) Cell lengths of the microscopy evaluation are shown and expressed as mean ± sdv and T/UT = treated/untreated; *** = *p* < 0.001 (S-FZ95 res. #2 used as reference); N ≥ 100 cells from 2 independent cultures. (**C**) The same strains, together with WM1074 as a control, were serially diluted and spotted on LB plates ± 0.5 μg/mL (*S*)-FZ95 and incubated overnight at 37 °C. (**D**) Swarm plot representing cell length values for each culture; N ≥ 100 cells from 2 independent cultures.

Because the causes of resistance in strain #1 remained partially unclear, we then asked whether FtsW_Q221L_ might have a role in conferring resistance in strain #2, given the importance of FtsW in cell division. Synthesis of septal peptidoglycan by FtsW occurs later in the cell division cycle than FtsZ ring assembly, and recruitment of FtsW to the cell division machinery depends on FtsZ [9]. However, it is possible that alteration of FtsW might feed back onto the FtsZ ring or bypass an inhibited pathway, potentially affecting BDOB resistance. To test if FtsW_Q221L_ might be responsible for the resistant phenotype, we replaced it with the WT *ftsW* allele in strain #2 following the same strategy described before, but using Δ*ilvI::kan*. The *ilvI* and *ftsW* genes are ~12 kb apart, resulting in a co-transduction frequency of ~65%. Also in this case, we first excised the Kan resistance cassette portion of the ∆*tolC::kan* marker in strain #2 through FLP recombination using pCP20. The Δ*ilvI::kan* tranductants were screened for either retention of the *ftsW*_*Q*221*L*_ allele or its conversion to WT *fts*W by sequencing the genomic copy of *ftsW*. We characterized one of each, naming them transductant #1 (WT *ftsW*, strain LS28) or transductant #2 (*FtsW*_Q221L_, strain LS29). Their phenotypes were tested once again by microscopy, following the experimental protocol described above with 0.5 μg/mL (S)-FZ95 (also see Section 4), and spotting serially diluted cells. Interestingly, transductant #1 carrying WT *ftsW* was significantly more susceptible to (*S*)-FZ95 compared to both resistant strain #2 and transductant #2 expressing FtsW_Q221L_. Specifically, transductant #1 cells (Figure 10A) show a T/UT ratio of 3.16, comparable to the value of 4.87 measured for the WM6922 parent (Figure 10B). Conversely, transductant #2, expressing the variant FtsW_Q221L_, shows a T/UT ratio almost identical to (*S*)-FZ95 res. strain #2, from which it originated (Figure 10B). The same phenotype was observed on solid media (Figure 10C).

To test whether *ftsW*_*Q*221*L*_ might be sufficient for the (*S*)-FZ95 resistance of strain #2, we replaced the chromosomal WT *ftsW* gene with *ftsW*_*Q*221*L*_ in a Δ*tolC* background strain. To prepare this strain, we first removed the Kan resistance cassette by FLP recombination using pCP20 from WM6922 (WM1074 Δ*tolC::kan*), then introduced *ftsW*_*Q*221*L*_, which should be ~75% cotransducible with the Δ*ilvI::kan* marker 12 kb away, through P1 transduction. We screened for either retention of WT *ftsW* (LS31) or conversion to the *ftsW*_Q221L_ allele (LS32) by sequencing DNA around the *ftsW* locus. Similar to the previously described strategy, the phenotypes of these strains were studied through microscopy and spotting serially diluted cells. As expected, LS32 had a 1000-fold increased viability on plates compared to LS31 in the presence of 0.25 or 0.50 μg/mL (*S*)-FZ95 (Figure S3B), and LS32 cells divided significantly better (T/UT ratio 1.59 vs. 3.54, Figure S3A,C). All together, these data suggest that the FtsW_Q221L_ variant contributes significantly to the (*S*)-FZ95 resistance in strain #2.

### 2.6. FtsW* Mutations (Including ftsW_Q221L_) Provide Resistance Towards Selected BDOBs

We next asked how *ftsW*_*Q*221*L*_ might promote resistance to (*S*)-FZ95. FtsW, together with FtsI, is recruited after early divisome proteins, and its recruitment is mediated by FtsQ, FtsL, and FtsB (FtsQLB complex) [9]. The FtsWI complex co-localizes with PBP1b, forming a ternary complex, which upon activation by another divisome protein, FtsN, is responsible for both glycan polymerization and cross-linking [34]. As previously reported, the hyperfission variant FtsW_E289G_ (also defined as FtsW*) is self-activated and thus no longer dependent on FtsN for divisome activation [35]. Given its ability to confer resistance to FZ95, we hypothesized that FtsW_Q221L_ might have FtsW*-like properties.

**Figure 11:**
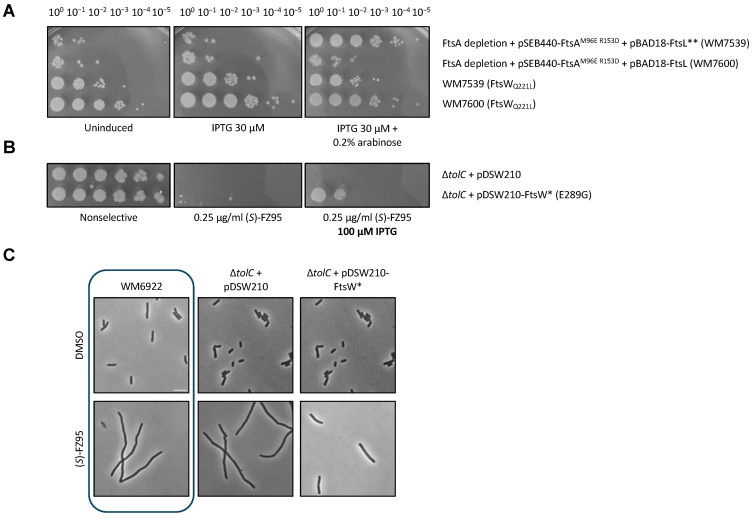
FtsW_Q221L_ is a hyperfission FtsW* variant similar to FtsW_E289G_. (**A**) To test if FtsW_Q221L_ can suppress the inability of FtsA_M96E R153D_ to form double-stranded filaments, FtsA depletion strains WM7539, WM7600, WM7539 FtsW_Q221L_ (LS33), and WM7600 FtsW_Q221L_ (LS34) were serially diluted and spotted on plates with no inducer, 30 μM IPTG to induce pSEB440-FtsA_M96E R153D_ or both 30 μM IPTG + 0.2% arabinose to induce pSEB440-FtsA_M96E R153D_, and pBAD18-FtsL (WT FtsL or FtsL**). The degree of growth on the spots correlates with the ability to bypass the defects of FtsA_M96E R153D_. (**B**) WM6922 (∆*tolC*) cells transformed with either pDSW210-FtsW_E289G_ (FtsW*) or empty vector were serially diluted and spotted onto LB plates with 0.25 μg/mL (*S*)-FZ95 or 0.25 μg/mL (*S*)-FZ95 + 100 μM IPTG to induce expression of the plasmid. (**C**) Cells were cultured twice at 30 °C until early-log phase (OD_600_ = 0.2/0.25), then induced with 100 µM IPTG for 1.5 h prior to imaging by phase contrast microscopy. Representative images are shown, with images of the parent ∆*tolC* cells boxed. Scale bar = 5 μm.

To test this idea, we asked whether FtsW_Q221L_ could suppress the defects of the FtsA_M96E R153D_ variant, which was recently shown to block the conversion of FtsA to antiparallel double filaments during septation. These FtsA filaments are specifically required for divisome activation but can be bypassed by hyperfission alleles such as FtsW* or FtsL** (FtsL_E88K G92D_) [36,37]. We first cotransduced the genomic *ftsW*_*Q*221*L*_ allele with the Δ*ilvI::kan* marker from LS27 into WM7539 and WM7600. These strains are capable of FtsA depletion when grown at 30 °C and carry pSEB440-FtsA_M96E R153D_, expressing the FtsA variant from an IPTG-dependent promoter, together with either pBAD18-FtsL (WM7600) or FtsL** (WM7539), expressed from arabinose-dependent promoters and serving as negative or positive controls, respectively. As expected, with both IPTG + arabinose under conditions of WT FtsA depletion, FtsL** allows FtsA_M96E R153D_ to function in cell division as the sole FtsA in the cell, serving as a positive control, whereas FtsL does not [36]. Importantly, FtsW_Q221L_ rescued FtsA_M96E R153D_ similarly to FtsL** (Figure 11A). Interestingly, the expression of both FtsL** and FtsW_Q221L_ confers less viability than FtsW_Q221L_ alone (Figure 11A, row 3), perhaps because of negative synergy between the two hyperfission alleles.

These data suggest that FtsW_Q221L_ is an FtsW*-like hyperfission variant similar to FtsW_E289G_. Consequently, following this logic, FtsW_E289G_ should be able to promote (*S*)-FZ95 resistance as well. To test this hypothesis, WM6922 (Δ*tolC*) was transformed with pDSW210-FtsW*, which expresses FtsW_E289G_ with IPTG, and this was tested with (*S*)-FZ95, comparing the results with the same strain transformed with the empty vector (LS35). We first spotted serially diluted cells onto 0.25 μg/mL (*S*)-FZ95 alone or with 100 μM IPTG added to induce FtsW* expression. As expected, WM6922 + pDSW210-FtsW* (LS36) was viable to the second dilution (Figure 11B), which is consistent with what we observed for FtsW_Q221L_ transduced into WM6922 (Figure S3). Secondly, we evaluated the cell morphology of these strains in the presence or absence of (*S*)-FZ95, following the previously described protocol. As predicted, LS36 cells filamented significantly less than those of LS35 in the presence of 0.5 μg/mL (*S*)-FZ95 (Figure 11C).

## 3. Discussion

In this work, we explore the effects of BDOBs on the Gram-negative model bacterium *Escherichia coli* and confirm that the presence of efflux pumps remains the main obstacle to the effectiveness of BDOBs against *E. coli* and likely other Gram-negative bacteria. Importantly, we demonstrate that BDOBs interact with *E. coli* FtsZ IDC to promote their antimicrobial activity, but we also observed how other non-FtsZ and non-efflux pump-related factors can suppress the antimicrobial activity of BDOBs, including hyperfission variants of the essential septal transglycosylase FtsW.

The utility of benzamides as potential drugs against Gram-negative bacterial infections has been controversial. While recent reports have shown that some benzamide derivatives such as TXY6129 possess antimicrobial activity against Gram-negative species in the presence of efflux pump inhibitors, other BDOBs do not have an antimicrobial effect even under these conditions [12]. Moreover, several research groups showed that other benzamides, such as PC190723, might not interact with *E. coli* FtsZ at all [24,25,26]. Given the increasing development of efflux pump inhibitors as antimicrobial adjuvants [38], the ability of BDOBs to exert their action when co-administered with PAβN supports the potential preclinical and clinical development of this class of compounds against Gram-negative targets.

**Figure 12:**
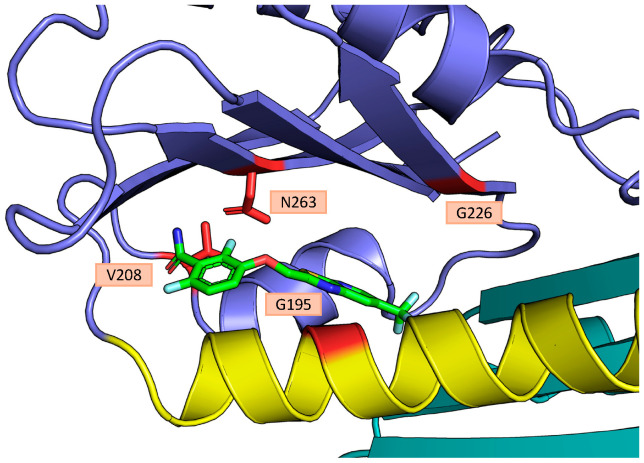
Position of relevant amino acids for benzamide binding within the *E. coli* FtsZ IDC.

Herein, our isolation of FZ101/(*S*)-FZ95-resistant mutants mapping to the IDC of *E. coli* FtsZ that are sufficient for resistance strongly suggests that FtsZ is the target of BDOBs in *E. coli* cells. Moreover, the isolation of spontaneously resistant mutants with modifications at residue N263 suggests that, despite some residue differences between Gram-positive and Gram-negative FtsZs, BDOBs retain the ability to interact with the IDC across species, as has been hypothesized but never demonstrated in *E. coli* so far. Mutations at N263 seem to be the most common way for Gram-positive bacteria to escape the antimicrobial action of benzamides, especially N263K [30]. On the contrary, these mutations in *E. coli* FtsZ significantly impair functionality (albeit not completely) and therefore represent a less viable option compared to Gram-positives. The isolation of a spontaneously resistant mutant expressing the FtsZ_G226V_ variant is further evidence that the IDC is the target in *E. coli*. Considering its position relative to the IDC, it is likely that changing G226 to a bulkier residue such as valine sterically impedes drug access to the binding site, similarly to what was described for G196 in *S. aureus* and *B. subtilis*. Indeed, *E. coli* FtsZ residue G195, which corresponds to G196 of *S. aureus* and *B. subtilis*, sits on the opposite side of the IDC from G226 (Figure 12), and its alteration also confers partial BDOB resistance in *E. coli*. Lastly, the partially resistant phenotype of engineered mutations such as V208A serves as an additional validation of this model. We modeled *E. coli* FtsZ bound to TXA707 (which showed activity against *E. coli* N43 [12]), using PHYRE2, by threading its amino acid sequence onto the available crystal structure of *S. aureus* FtsZ bound to TXA707 (PDB 5xdt). In this model, all the residues mentioned are predicted to be near the TXA707 binding site or at the entrance of the cleft, as postulated previously (Figure 12).

How then, to explain the inability of PC190723 to interact with *E. coli* FtsZ or of other benzamides to exert antimicrobial activity in the presence of efflux-pump inhibitors? We speculate that compounds such as FZ100 and FZ95, with known high affinities for *E. coli* FtsZ in vitro [11], could bind FtsZ strongly enough to disrupt the site-occluding non-covalent interactions recently reported [25], effectively inhibiting FtsZ activity.

The general mechanism of action of benzamides is well characterized. In Gram-positive species such as *B. subtilis*, benzamides rapidly block cell division, with FtsZ redistribution to subcellular foci and consequent cell filamentation. It was also demonstrated how disassembled and inactive FtsZ retains the ability to recruit late divisome proteins, although the cell division process is no longer able to occur. This effect was correlated with FtsZ polymer hyperstabilization in vitro, coupled with reduced GTPase activity [39]. In Gram-negative species such as *E. coli*, we previously demonstrated how BDOBs behave similarly, inhibiting FtsZ depolymerization and the GTPase activity of the protein in vitro [11]. In contrast to *B. subtilis*, we observed that in *E. coli* exposed to BDOBs, most Z rings do not convert to foci but instead appear to be locked in place as rings, similar to Z rings in *E. coli* cells exposed to PC190723 observed previously [24]. Consistent with that previous report, the filamentation of cells treated with BDOB indicates that these Z rings in *E. coli* are unable to complete septation.

Other than modifications to the FtsZ target, different mutations conferring resistance to benzamides have been reported. For example, several non-*ftsZ* spontaneous mutants conferred resistance to PC190723 in *E. coli* [24], although their mechanisms of action remain unknown. In the present work, we also found mutant alleles of two genes, *sanA* and *yidE*, not reported previously, that confer some degree of resistance to FZ95. The mechanism behind the effects of the mutant *yidE* allele is unclear, but there are some clues for the effect of the mutant *sanA* allele in resistant strain #1. Inactivation of *sanA* in *E. coli* was recently reported to suppress multiple defects in later stages of cell division, including defects in *ftsI*, and enhanced overall synthesis of peptidoglycan [40], probably by increasing lipid II availability. These effects might mimic FtsW* and divisome activation, which is a property of resistant strain #2 (see below). On the other hand, inactivation of *sanA* also increases membrane permeability [41]; if the *sanA* allele we isolated is a loss-of-function allele, it is hard to understand how increased membrane permeability would lead to BDOB resistance. The caveat here is that the clean deletion of sanA did not confer BDOB resistance, so the mechanism is still unclear.

Nonetheless, our isolation of a hyperfission variant of FtsW that confers a modest FZ95 resistance phenotype on its own sheds new light on the mechanism of benzamide action. Based on previous models, in the presence of WT FtsW, BDOBs bind and inactivate *E. coli* FtsZ, “freezing” the Z rings in an inactive form, probably by rapidly altering FtsZ treadmilling activity at the membrane as was observed with *B. subtilis* FtsZ [42]. Without proper Brownian ratchet activity from treadmilling, FtsZ is unable to assemble other essential divisome proteins, including FtsW, into an active septum-synthesis complex [43]. The ability of FtsW* variants such as FtsW_Q221L_ or FtsW_E289G_ to partially suppress this inhibition indicates that the defective Z rings can still recruit divisome proteins such as FtsW in the presence of FZ95 but fail to activate septum synthesis. Therefore, we speculate that FZ95 specifically affects FtsZ’s ability to promote FtsWI activity, potentially by inhibiting the conversion of FtsA to the antiparallel double-stranded filament form [36,44]. It is notable that the location of Q221L on the FtsW molecule is predicted to be cytoplasmic, in the third cytoplasmic loop between transmembrane helices 6 and 7, whereas previously reported *E. coli* FtsW* variants, E289G and M269I, are both located in the fourth extracellular loop (ECL4) [45]. The strongest FtsW* allele outside of *E. coli* is A246T of *Caulobacter crescentus*, also in ECL4 at the same position as M269I. Weaker FtsW* alleles in *C. crescentus* FtsW include T180A (ECL3) and F145L (cytoplasmic loop 2) [46]. Previous dominant negative alleles mapping to cytoplasmic loop 2 of *E. coli* FtsW suggested that FtsA acts on cytoplasmic loop 2 of FtsW to activate FtsWI and consequently septum synthesis [45]. In light of this, we propose that the Q211L change in the adjacent cytoplasmic loop 3 of FtsW mimics activation of FtsW by FtsA on the cytoplasmic side, much as the M269I and E289G changes mimic activation of FtsW by FtsN on the periplasmic side [37]. Although FtsW_E289G_ is a strong FtsW* allele capable of bypassing significant defects in divisome activation, its relatively weak resistance to FZ95 on its own is similar to that of FtsW_Q211L_. This emphasizes the importance of other non-FtsZ factors such as membrane permeability and the role of proteins such as SanA in mediating resistance to BDOBs and likely other compounds that inhibit bacterial cell division.

In addition to their use as tools to investigate the molecular mechanisms of bacterial cell division, BDOBs continue to hold promise as antimicrobials against Gram-negative as well as Gram-positive infections. Indeed, the high antimicrobial potencies of specific derivatives against efflux pump deficient (or inhibited) *E. coli* strains are comparable to those against Gram-positive bacteria. The recently demonstrated potent effects of benzamide compounds against *A. baumannii* [12] support this premise.

## 4. Materials and Methods

### 4.1. Strains, Plasmids, and Growth Conditions

All strains and plasmids used for this study are described in Table S2 in the Supplementary Information. Bacterial cultures were grown with Lennox lysogeny broth (LB) or agar plates unless otherwise indicated. Strains containing plasmids were supplemented as needed with ampicillin (50 μg/mL), chloramphenicol (15 μg/mL), kanamycin (25 μg/mL), IPTG (various concentrations), or L-arabinose (0.2%) (MilliporeSigma, St. Louis, MO, USA). Optical density of liquid cultures was assessed at 600 nm (OD600). The strain for plasmid construction was XL1-Blue. *E. coli* transformation and DNA manipulations were carried out using standard methods, verifying each step through DNA sequencing. Statistical analyses (one-way ANOVA) were carried out with GraphPad Prism (10.4.1), always using CI = 95% and 2 independent biological replicates.

### 4.2. Construction of Plasmids and Strains

All plasmids are listed in Table S2. All modifications of the *ftsZ* gene (M206F, V208A, M206F, and V208A, G195A, N263K, G226V, N263Y, and M206V) were constructed by site-directed mutagenesis using primers 2865 + 2866, 2867 + 2868, 2873 + 2874, 2869 + 2870, 2871 + 2872, 2899 + 2900, 2901 + 2902, and 2915 + 2916, respectively. All primers used for this study are listed in Table S3. To construct Δ*yidE::kan*, Δ*tolC::kan,* Δ*dld::kan,* and Δ*ilvI::kan* strains, the respective Keio collection strains were cultured to make a P1 phage lysate carrying the corresponding allele and subsequently transduced into the proper recipient strain. Transductants were then streak purified and saved. The gene insertion was verified through colony PCR gene amplification and either size comparison on 1% agarose gel between donor and receiving strains or sequencing, as specifically described in the main text.

### 4.3. MIC Assays with PAβN

The MIC evaluation was carried out similarly to what was previously described [11]. Briefly, 1 μL of a 30 mg/mL compound stock solution (either FZ101 or (*S*)-FZ95) was added to 500 μL of LB, mixed carefully, and 100 μL of this was added to the first well of a 96-well microplate. Then, 50 μL were transferred from the first well to the second, which contained 50 μL of LB, and so on until the eighth well, resulting in a serial 1:2 dilution of the concentration. Then, mid-log WM1074 cells were diluted 1:300 in fresh LB or in a 200 μg/mL solution of PAβN (MP Biomedicals, Irvine, CA, USA) solution in LB, when appropriate. Lastly, 50 μL of cells were added to each well, resulting in final compound concentrations of 30, 15, 7.5, 3.75, 1.87, 0.94, 0.47, and 0.24 μg/mL, respectively, and in a final PAβN concentration of 100 μg/mL, when present. As a negative control, 1 μL of DMSO was added in place of the compound stock solution and then subsequently treated as described. The microplate was then incubated overnight at 37 °C with shaking, and turbidity in each well was measured with an Agilent Biotek Synergy H1 microplate reader (Santa Clara, CA, USA) at 600 nm wavelength every 15 min. The MIC was determined as the lowest concentration at which no OD increase was observed after overnight incubation.

### 4.4. Spontaneous Mutant Screening

*E. coli* Δ*tolC*::*kan* cells (WM6922) were incubated overnight at 30 °C. Then, 100 μL of cells were spread on plates containing 15 μg/mL (4XMIC) of FZ101 and incubated again at 30 °C for 24 h. Resistant candidates were streaked again onto FZ101 plates and non-selective and incubated at 30 °C overnight to confirm the resistant phenotype. Mutations in chromosomal *ftsZ* were identified by DNA sequencing using primers 2401 and 2402.

### 4.5. Phenotypic Evaluation of Drug-Resistant Strains

Cells of the investigated strain were cultured to saturation at 30 °C or 37 °C, as specified in the main text. Then each strain was cultured in two replicates with the opportune plasmid inducer and antibiotic for selection, when opportune, until early-log phase (OD = 0.2/0.25). Then, one of the replicates was added with the BDOB in evaluation ((*S*)-FZ95 or FZ101) and the other with a corresponding volume of DMSO. Cells were grown again at the same temperature for 1 h prior to OD measurement and microscopy evaluation.

### 4.6. FtsZ Mutant Functionality Assay

WM5188 (WM1074 *ftsZ84*ts Δ*tolC*::*kan*) was transformed with plasmids encoding either wild-type FtsZ or one of the engineered FtsZ mutants (M206F, V208A, M206F and V208A, G195A, N263K, M206V). Each strain was cultured at 30 °C with 0.2% arabinose until the early-log phase (OD = 0.2/0.25), then switched to 42 °C for 1 h prior to microscopic visualization.

### 4.7. Spot Viability Assays

Cells for each strain were cultured to mid-exponential phase in LB prior to 10-fold serial dilution in separate rows of a microtiter plate. BDOB-containing plates were prepared as previously described [11]. Dilutions were spotted using a 48-pin cell replicator, and plates were incubated at 37 °C overnight.

### 4.8. Microscopy

Cell suspensions were mounted on agarose pads on glass slides, covered with a #1.5 coverslip, and imaged on an Olympus BX63 microscope (Tokyo, Japan) under a 100X oil objective (N.A. 1.4) equipped with a Hamamatsu C11440 ORCA-Spark digital complementary metal oxide semiconductor (CMOS) camera (Hamamatsu Photonics K.K., Hamamatsu, Japan) using cellSens Dimension 4.2 software (Olympus). Images were analyzed using Fiji/ImageJ (2.14.0)/MicrobeJ (5.13p) [47,48]. When cell lengths were measured, at least 100 cells from 2 independent biological replicates were analyzed. Statistical analyses were carried out with GraphPad Prism (10.4.1).

## Figures and Tables

**Table 1:** Evaluation of (*S*)-FZ95 and FZ101 against *E. coli* in combination with PAβN (100 μg/mL).

	MIC (μg/mL)		
	*E. coli* Wt		*E. coli* Δ*tolC*
Compound	Alone	+PaβN	Alone
(*S*)-FZ95	>30	0.47	0.47
FZ101	>30	7.5	7.5

**Table 2:** Genome comparison between (*S*)-FZ95 res. strains #1 and #2.

	(*S*)-FZ95 Res. Strain #1	(*S*)-FZ95 Res. Strain #2
*yidE*	Deletion of G at position 131	Deletion of G at position 131
*sanA*	1342 bp insertion at position 495	*Unmodified*
*ftsW*	*Unmodified*	SNP A to T at position 812

## Data Availability

The original contributions presented in this study are included in the article/Supplementary Material. Further inquiries can be directed to the corresponding author.

## Supplementary Materials

The following supporting information can be downloaded at: https://www.mdpi.com/article/10.3390/antibiotics15020126/s1, Figure S1: G226V and N263Y FtsZ variants confer resistance against (*S*)-FZ95; Figure S2: FZ101-resistant FtsZ variants promote different degrees of resistance against (*S*)-FZ95; Figure S3: Replacement of the native *ftsW* gene with *ftsW*_*Q*221*L*_ in the Δ*tolC* ((*S*)-FZ95-sensitive) parent strain provides partial (*S*)-FZ95 resistance; Table S1: OD_600_ values of WM6922 and (*S*)-FZ95 resistant strain overnight cultures, grown either in 1 μg/mL (*S*)-FZ95 or plain LB. Data are representative of 2 independent replicates; Table S2: Strains and plasmids used in this study; Table S3: Oligonucleotide primers used in this study [36,49,50].
