# Supplementary material for: Mechanistic Insights into the Antimicrobial Effect of Benzodioxane-Benzamides Against Escherichia coli
Source: Antibiotics (Basel). 2026 Jan 27;15(2):126. doi: 10.3390/antibiotics15020126 (PMC12937226; doi:10.3390/antibiotics15020126)
Supplement: Supplementary file 1 [file antibiotics-15-00126-s001.zip › antibiotics-4074510-supplementary.pdf]

SUPPLEMENTARY MATERIALS

Mechanistic insights into the antimicrobial effect of benzodioxane-benzamides against *Escherichia coli*

Lorenzo Suigo[1], Alessia Lanzini[2], Valentina Straniero[2], William Margolin[1,*]

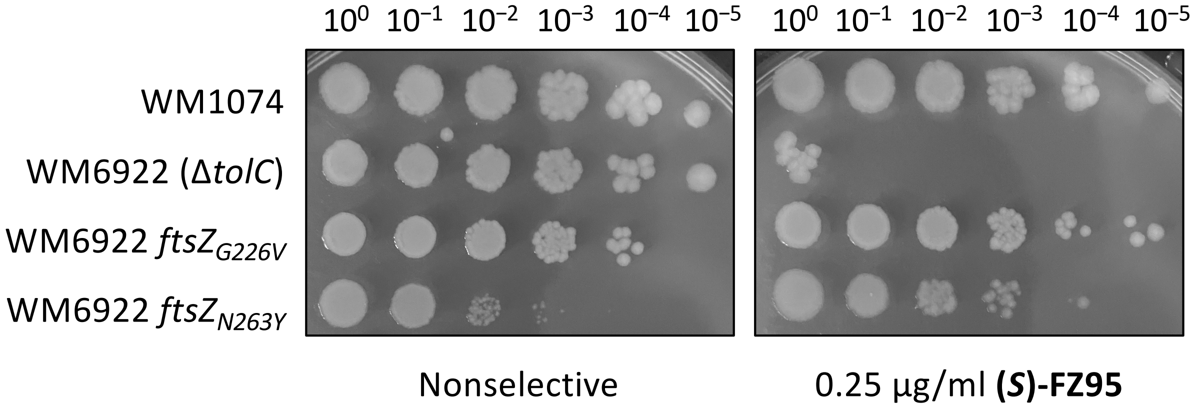

Fig. S1. G226V and N263Y FtsZ variants confer resistance against (*S*)-FZ95. Growth of serially diluted WM1074, WM6922 *(*Δ*tolC*), WM6922 $FtsZ_{G226V}$ and WM6922 $FtsZ_{N263Y}$ on LB plates ± 0.25 µg/mL (*S*)-FZ95.

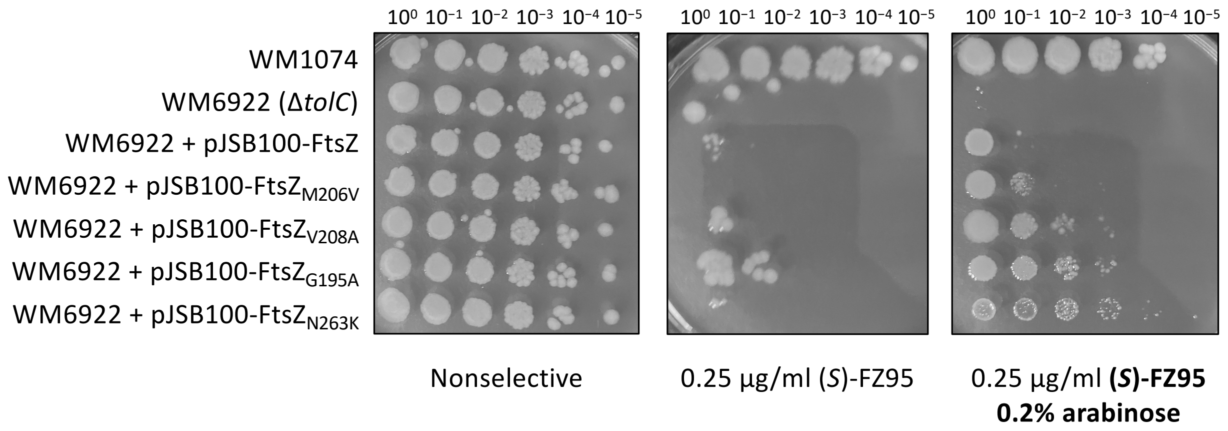

Fig. S2. FZ101 resistant FtsZ variants promote different degrees of resistance against (*S*)-FZ95. Growth of serially diluted WM1074, WM6922 and WM6922 transformed with pJSB100 plasmids expressing each FtsZ variant shown on LB plates containing (*S*)-FZ95 ± 0.2% arabinose to induce FtsZ expression.

A

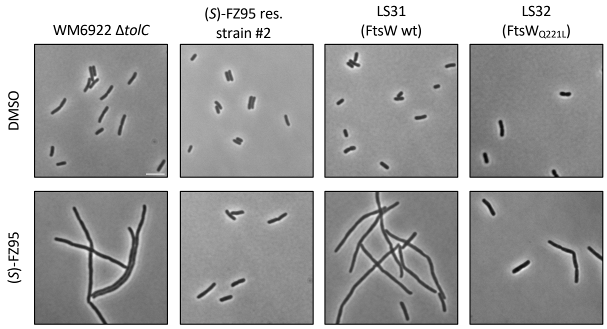

B

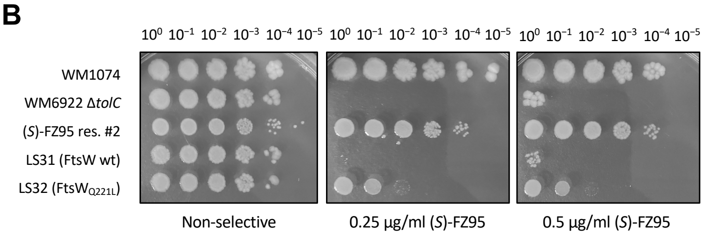

C

	(*S*)-FZ95	DMSO	T/UT Ratio	Phenotype
WM6922	14.70 ± 8.5	3.47 ± 0.9	4.24	Sensitive
(*S*)-FZ95 res. #2	3.92 ± 1.1	2.65 ± 0.5	1.48	Resistant
LS31 (wt)	11.04 ± 6.1	3.11 ± 0.9	3.54	Sensitive
LS32 (Q221L)	4.68 ± 2.4*	2.94 ± 0.7	1.59	Intermediate

Fig. S3. Replacement of the native *ftsW* gene with *ftsW*$_{Q221L}$ in the *ΔtolC* ((*S*)-FZ95-sensitive) parent strain provides partial (*S*)-FZ95 resistance. (A) As previously described, the *E. coli* Δ*tolC* parent (WM6922), (*S*)-FZ95 res. strain #2, LS31 (expressing FtsW WT) and LS32 (expressing FtsW$_{Q221L}$) were cultured twice at 30˚C until early-log phase (OD_{600} = 0.2/0.25), then one culture per strain was treated with 0.5 µg/mL (*S*)-FZ95 and the other with a corresponding volume of DMSO. Cells were grown for 1.5 h prior to imaging by phase contrast microscopy. Representative images are shown. Scale bar = 5 µm. (C) Cell lengths from the microscopic evaluation are shown and expressed as mean ± sdv. T/UT = treated/untreated. * = $P < 0.05$ (S-FZ95 res. #2 used as reference); N ≥ 100 cells from 2 independent cultures. (D) The same strains, together with WM1074 as a control, were serially diluted and spotted on LB plates ± 0.5 µg/mL (*S*)-FZ95 and incubated overnight at 37˚C.

Table S1. OD_{600} values of WM6922 and (*S*)-FZ95 resistant strain overnight cultures, grown either in 1 µg/mL (*S*)-FZ95 or plain LB. Data are representative of 2 independent replicates.

**Strain**	**LB**	**(*S*)-FZ95**
WM6922	6.40	2.99
(*S*)-FZ95 res. #1	5.80	4.20
(*S*)-FZ95 res. #2	5.90	4.56
(*S*)-FZ95 res. #8	6.77	3.93
(*S*)-FZ95 res. #9	5.15	1.80
(*S*)-FZ95 res. #11	6.67	4.47

Table S2. Strains and plasmids used in this study.

Strain	Relevant genotype	Source/Reference
XL1-Blue	Cloning strain	Lab collection
JW3662	*ΔyidE::kan*, Keio strain	Nick De Lay
JW0076	*ΔilvI::kan*, Keio strain	Nick De Lay
JW2121	*Δdld::kan*, Keio strain	Nick De Lay
WM1074	MG1655 *ΔlacU169*	Lab collection
WM5188	WM1074 *ftsZ84 ΔtolC::kan*	Lab collection
WM6794	N43 (*acrA*-), efflux pump defective	[1]
WM6922	WM1074 *ΔtolC::kan*, efflux pump defective	Lab collection
WM4952	MT78 (*leuA*::*Tn10 FtsA^{0}*, *zapA*-GFP, pSC101-P_R-*FtsA*	[2]
WM7539	WM4952 + pSEB440-$FtsA_{M96E\ R153D}$ + pBAD18-FtsL**	[3]
WM7600	WM4952 + pSEB440-$FtsA_{M96E\ R153D}$ + pBAD18-FtsL	[3]
LS01	WM6922 $FtsZ_{G226V}$	This study
LS02	WM6922 $FtsZ_{N263Y}$	This study
LS03	WM5188 + pJSB100-$FtsZ_{G226V}$	This study
LS04	WM5188 + pJSB100-$FtsZ_{N263Y}$	This study
LS05	WM6922 + pJSB100-$FtsZ_{G226V}$	This study
LS06	WM6922 + pJSB100-$FtsZ_{N263Y}$	This study
LS07	WM5188 + pJSB100-$FtsZ_{M206F}$	This study
LS08	WM5188 + pJSB100-$FtsZ_{M206V}$	This study
LS09	WM5188 + pJSB100-$FtsZ_{V208A}$	This study
LS10	WM5188 + pJSB100-$FtsZ_{M206F\ V208A}$	This study
LS11	WM5188 + pJSB100-$FtsZ_{G195A}$	This study
LS12	WM5188 + pJSB100-$FtsZ_{N263K}$	This study
LS13	WM6922 + pJSB100-$FtsZ_{M206V}$	This study
LS14	WM6922 + pJSB100-$FtsZ_{V208A}$	This study
LS15	WM6922 + pJSB100-$FtsZ_{G195A}$	This study
LS16	WM6922 + pJSB100-$FtsZ_{N263K}$	This study
LS17	(*S*)-FZ95 resistant strain #1	This study
LS18	(*S*)-FZ95 resistant strain #2	This study
LS19	(*S*)-FZ95 resistant strain #8	This study
LS20	(*S*)-FZ95 resistant strain #9	This study
LS21	(*S*)-FZ95 resistant strain #11	This study
LS22	WM1074 *ΔyidE*	This study
LS23	WM1074 *ΔyidE ΔtolC*::*kan*	This study
LS24	WM6922 + pDSW210-*yidE*	This study
LS25	WM6922 + pDSW210-*yidE**	This study
LS26	(*S*)-FZ95 res. strain #1 Δ*dld::kan sanA* mutated	This study
LS27	(*S*)-FZ95 res. strain #1 Δ*dld::kan sanA* WT	This study
LS28	(*S*)-FZ95 res. strain #2 Δ*ilvI::kan ftsW*	This study
LS29	(*S*)-FZ95 res. strain #2 Δ*ilvI::kan* $ftsW_{Q221L}$	This study
LS30	WM6922 *ΔtolC* (kan cassette removed)	This study
LS31	WM6922 *ΔtolC ΔilvI::kan* (FtsW WT)	This study
LS32	WM6922 *ΔtolC ΔilvI::kan* ($FtsW_{Q221L}$)	This study

LS33	WM7539 $FtsW_{Q221L}$	This study
LS34	WM7600 $FtsW_{Q221L}$	This study
LS35	WM6922 + pDSW210	This study
LS36	WM6922 + pDSW210-FtsW*	This study
**Plasmid**	**Description**	**Source**
pJSB100-$FtsZ_{M206F}$	pJSB100 expressing $FtsZ_{M206F}$, arabinose control	This study
pJSB100-$FtsZ_{V208A}$	pJSB100 expressing $FtsZ_{V208A}$, arabinose control	This study
pJSB100-$FtsZ_{M206F\ V208A}$	pJSB100 expressing $FtsZ_{M206F\ V208A}$, arabinose control	This study
pJSB100-$FtsZ_{G195A}$	pJSB100 expressing $FtsZ_{G195A}$, arabinose control	This study
pJSB100-$FtsZ_{N263K}$	pJSB100 expressing $FtsZ_{N263K}$, arabinose control	This study
pJSB100-$FtsZ_{N263Y}$	pJSB100 expressing $FtsZ_{N263Y}$, arabinose control	This study
pJSB100-$FtsZ_{G226V}$	pJSB100 expressing $FtsZ_{G226V}$, arabinose control	This study

Table S3. Oligonucleotide primers used in this study.

Primer #	5'-3' nucleotide sequence
2401	CTCGTGGATCAAGCGACTCA
2402	AGTGTCCTTTGTTTGATCATCGT
2865	TCCGGGTTTGTTTAACGTGGACTTTG
2866	CGAGTAATCAGTTCAGCG
2867	TTTGATGAACGCGGACTTTGCAG
2868	CCCGGACGAGTAATC
2869	CGCTGTGCAAGCGATCGCTGAAC
2870	CCTTTCAGTACATCGTTCG
2871	CGTGCTGGTTAAAATCACGGCGG
2872	CCGCGCGCGCCAGAC
2873	CGCCGACTTTGCAGACGTACGC
2874	TTAAACAAACCCGGACGAGTAATC
2899	GCTACGCAATGATGGTTTCTGGCGTGGCGAG
2900	CTCGCCACGCCAGAAACCATCATTGCGTAGC
2901	GCGGCGTGCTGGTTTATATCACGGCGGGCT
2902	AGCCCGCCGTGATATAAACCAGCACGCCGC
2915	TCCGGGTTTGGTGAACGTGGACT
2916	CGAGTAATCAGTTCAGCGATACC
2974	AAACAGTTTGCCGTTTCTTG
2975	TTCGCGGAAACAAAATATCG

Supplementary references

(1) Nakamura, H.; Suganuma, A. Membrane Mutation Associated with Sensitivity to Acriflavine in *Escherichia Coli*. *J. Bacteriol.* **1972**, *110* (1), 329–335. https://doi.org/10.1128/jb.110.1.329-335.1972.

(2) Tsang, M.; Bernhardt, T. G. A Role for the F Ts QLB Complex in Cytokinetic Ring Activation Revealed by an *Fts L* Allele That Accelerates Division. *Mol. Microbiol.* **2015**, *95* (6), 925–944. https://doi.org/10.1111/mmi.12905.

(3) Perkins, A.; Mounange-Badimi, M. S.; Margolin, W. Role of the Antiparallel Double-Stranded Filament Form of FtsA in Activating the *Escherichia Coli* Divisome. *mBio* **2024**, *15* (8), e01687-24. https://doi.org/10.1128/mbio.01687-24.
